# THE FACTORS RELATED TO KENDO PRACTICE THAT AFFECT IKIGAI-KAN OF MIDDLE-AGED AND OLDER KENDO PRACTITIONERS

**DOI:** 10.1093/geroni/igad104.2448

**Published:** 2023-12-21

**Authors:** Daisuke Matsumoto, Hayato Uchida

**Affiliations:** University of Hyogo, Osaka-city, Osaka, Japan; University of Hyogo, Himeji-city, Hyogo, Japan

## Abstract

The purpose of this study was to clarify the factors related to kendo practice that affect Ikigai-Kan and actual conditions of generativity, mental health of middle-aged and elderly Kendo practitioners. We selected 97 middle-aged and older Kendo practitioners who are practicing at Dojos in Osaka and Hyogo prefectures as research participants. Items that are basic attributes were grouped into two groups, and the values of each survey item were compared between groups. In addition, correlation analysis was performed to grasp the mutual relationship between the basic attributes and each survey item value. Furthermore, in order to clarify the factors related to the practice of kendo that affect Ikigai-Kan, multiple regression analysis was performed. Each item value indicating the practice status of kendo was used as independent variable. As a result, it was confirmed that the frequency of practice with young people (β=0.384, p< 0.01) affected Ikigai-Kan. These results show that the collaboration between the school and the community, the creation of an environment where the skills and experience of middle-aged and elderly people such as club activity instructors and local sports organizers can be enhanced, and opportunities to interact with or practice with young people would lead to Ikigai.

